# Synergizing Radiotherapy and Immune Checkpoint Inhibitors in Malignant Solid Tumours: Mechanistic Insights and Translational Frontiers

**DOI:** 10.1017/erm.2026.10041

**Published:** 2026-03-10

**Authors:** Jiahui Dai, Lingwei Ma, Xinyi Han, Xiong Li, Lingfei Han, Wei Wang

**Affiliations:** 1 First Affiliated Hospital of Guangzhou Medical University, China; 2 Tongji University Shanghai First Maternal and Infant Hospital, China

**Keywords:** abscopal effect, combination therapy, cGAS-STING pathway, immune checkpoint inhibitors, immunogenic cell death, radiotherapy, translational oncology, tumour immune microenvironment

## Abstract

**Background:**

Radiotherapy (RT) and immune checkpoint inhibitors (ICIs) have each transformed the treatment of malignant solid tumors (STs). Beyond direct tumor killing, RT remodels the tumor microenvironment (TME), promotes antigen release, and enhances immune activation. ICIs targeting cytotoxic T-lymphocyte antigen 4 (CTLA-4), programmed cell death protein 1 (PD-1), and programmed cell death ligand 1 (PD-L1) restore antitumor immunity by reversing T cell exhaustion. Increasing evidence indicates that RT can synergize with ICIs through mechanisms such as the abscopal effect, immunogenic cell death (ICD), and activation of the cyclic guanosine monophosphate–adenosine monophosphate (cGMP–AMP) synthase–stimulator of interferon genes (cGAS–STING) pathway.

**Methods:**

This review summarizes current radiobiological, immunological, and clinical evidence regarding the synergistic effects of RT and ICIs in malignant STs, with a focus on underlying mechanisms, recent clinical advances, and translational challenges.

**Results:**

RT can enhance tumor immunogenicity, promote immune priming, and reshape the TME to improve the efficacy of ICIs. Synergy between RT and ICIs is associated with ICD induction, cGAS‒STING activation, enhanced systemic antitumor immunity, and modulation of immune cell infiltration and checkpoint signaling. Clinical studies across multiple STs have shown encouraging efficacy and manageable safety, although outcomes vary according to tumor type, disease stage, radiation schedule, and patient selection.

**Conclusions:**

RT combined with ICIs is a promising therapeutic strategy for malignant STs. Further optimization of treatment regimens and biomarker-guided patient selection will be essential to maximize clinical benefit and enable more precise combination therapies.

## Introduction

Malignant solid tumours (STs) remain a leading cause of morbidity and mortality worldwide, particularly in advanced and metastatic settings where conventional treatments often fail to achieve durable control. Radiotherapy (RT), one of the three cornerstones of oncologic management, is delivered to more than half of all patients with cancer and provides critical local tumour control (Refs 1, 2). Traditionally considered a purely locoregional modality, RT mediates its antitumour effects primarily through induction of DNA double-strand breaks (DSBs), mitotic catastrophe and cell-cycle arrest (Ref. 3).

Concurrently, the emergence of cancer immunotherapy – including therapeutic vaccines (Ref. 4), adoptive cell therapies (Ref. 5), cytokine treatments (Ref. 6) and, most notably, immune checkpoint inhibitors (ICIs) (Ref. 7) – has transformed the therapeutic landscape. ICIs have demonstrated durable clinical benefit in melanoma (Ref. 8), non-small cell lung cancer (NSCLC) (Ref. 9) and head and neck squamous cell carcinoma (HNSCC) (Ref. 10), among others. Yet, only a subset of patients benefits from ICIs monotherapy, largely due to immunosuppressive features of the tumour microenvironment (TME), such as the presence of regulatory T cells (Tregs), myeloid-derived suppressor cells (MDSCs) and hypoxia (Refs 11, 12). Overcoming the ‘immunologically cold’ tumour phenotype has emerged as a critical determinant for improving clinical outcomes.

RT has recently been recognized as more than a local cytotoxic therapy; it is a potent immunological modulator capable of orchestrating systemic antitumour immunity (Refs 13–15). Early-phase clinical studies have shown that RT can induce immunogenic cell death (ICD), enhance antigen presentation, promote infiltration of effector T cells and activate immune pathways such as the cyclic GMP-AMP synthase–stimulator of interferon genes (cGAS-STING) pathway (Refs 16–18). These processes can transform immunologically inert tumours into inflamed, immune-responsive phenotypes, thereby sensitizing them to checkpoint blockade.

The conceptual bridge between radiation and immune activation – first observed as the ‘abscopal effect’ – has since evolved from anecdotal curiosity to a mechanistically defined and clinically exploitable phenomenon. As the understanding of RT-induced immune modulation deepens, the combination of RT and ICIs has emerged as a frontier strategy in precision oncology, capable of integrating locoregional control with systemic immune surveillance.

This review delineates the theoretical underpinnings, mechanistic pathways and translational progress of combining RT with ICIs. By synthesizing radiobiological and immunological principles, we aim to provide a framework for optimizing combinatorial regimens, improving patient selection, and advancing combined RT and ICIs approaches.

## RT and ICIs

### The rise of major categories of RT

RT has remained one of the central pillars of cancer treatment for over a century, with continuous advancements in both technology and biological understanding. The evolution of RT can be broadly divided into four major eras – the discovery era, the orthovoltage era, the megavoltage era and the ion beam era – each representing a significant leap forward in clinical application and radiobiological insight (Refs 19, 20).

Advances in radiation physics from the 1930s onwards enabled the treatment of deep-seated malignancies and refined clinical protocols based on tissue tolerance and dose–response relationships (Refs 21–26). Notably, in 1953, Mole and colleagues made early observations of the abscopal effect, suggesting that RT could extend beyond local tumour control and possess the potential to induce systemic immune activation (Ref. 27).

Since the late 1980s, RT has entered the precision and ion beam era, integrating advanced imaging and computer-assisted planning through intensity-modulated radiotherapy (IMRT), image-guided radiotherapy (IGRT) and stereotactic body radiotherapy (SBRT), as well as proton radiotherapy (PRT) and carbon ion radiotherapy (CIRT), to achieve superior dose conformity and reduced toxicity (Refs 28–33). Beyond technical refinement, modern RT increasingly emphasizes its biological impact on the tumour immune microenvironment, providing the mechanistic foundation for the combination of RT and ICIs.

### The evolution of ICIs

The discovery of immune checkpoint pathways has fundamentally reshaped cancer immunotherapy by revealing how tumours exploit physiological immune tolerance mechanisms to evade immune surveillance (Refs 34–37).

A transformative breakthrough emerged in the early 21st century with the development of ICIs targeting cytotoxic T lymphocyte antigen-4 (CTLA-4) (Ref. 38), programmed cell death 1 (PD-1) (Ref. 39) and programmed cell death ligand 1 (PD-L1) (Refs 40, 41). These agents reverse inhibitory immune signalling and enable sustained antitumour T cell responses, leading to unprecedented survival benefits across multiple STs (Refs 42–44). The success of ICIs not only validated the therapeutic potential of immune modulation but also reshaped the landscape of oncology.

In addition to these classical checkpoints, several emerging inhibitory pathways, such as lymphocyte activation gene 3 (LAG-3) and T cell immunoreceptor with Ig and ITIM domains (TIGIT), have recently entered clinical investigation, further expanding the immune checkpoint landscape (Refs 45–48).

### The rise of the combination of RT and ICIs

The integration of RT and ICIs represents a major conceptual shift in contemporary oncology. While each has long been a cornerstone in the management of malignant STs, their combination has evolved from theoretical speculation to clinical reality, establishing a new paradigm for systemic cancer control.

The conceptual link between radiation and immune modulation was first recognized in 1953, when Mole described the ‘abscopal effect’ – the regression of distant, non-irradiated tumours following local irradiation – suggesting an immune-mediated mechanism (Ref. 27).

The modern phase of integrating RT with ICIs began with the recognition that RT can function as an in situ tumour vaccine by promoting antigen release and immune activation (Ref. 49). The subsequent clinical success of ICIs rapidly accelerated the translation of this combination strategy into clinical trials across multiple STs.

Mechanistically, RT induces ICD, enhances tumour antigen presentation, activates the cGAS-STING pathway and promotes cytotoxic T cell infiltration, collectively converting immunologically ‘cold’ tumours into ‘hot’ ones and sensitizing tumours to ICIs (Refs 50, 51).

Beyond classical immune checkpoints, emerging evidence suggests that RT may also synergize with next-generation ICIs targeting alternative exhaustion pathways, such as LAG-3 and TIGIT (Refs 52–54). Agents including relatlimab and tiragolumab have shown clinical activity in early studies, highlighting the future potential of RT-based immune priming in next-generation checkpoint strategies.

Together, these developments establish RT as a key immunomodulatory partner for ICIs and provide the biological foundation for future RT and ICIs combination approaches.

## Mechanistic basis of the combination of RT and ICIs

**Figure 1.**
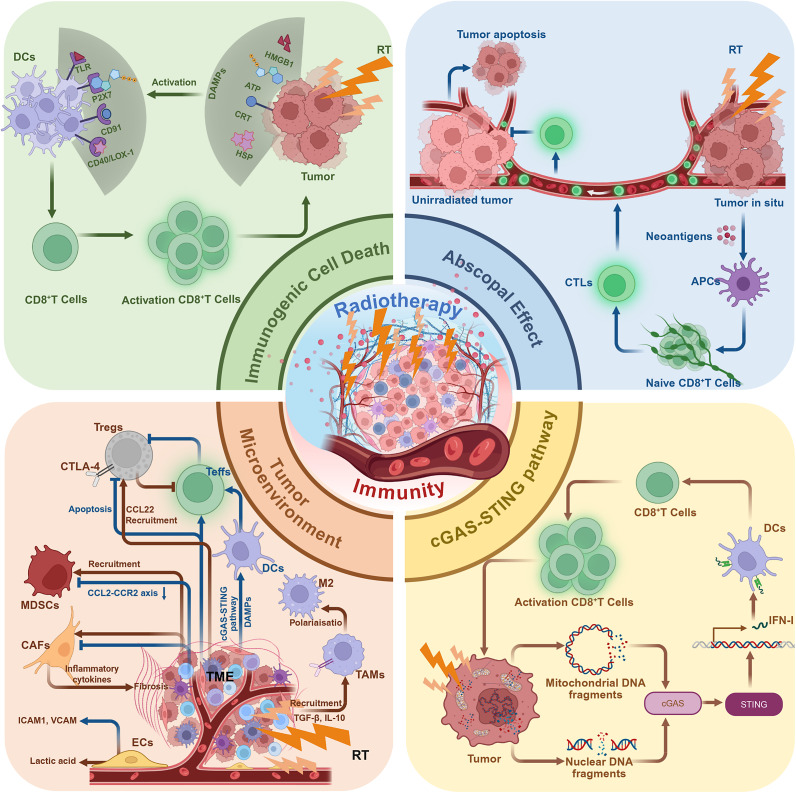
Mechanistic synergy between RT and ICIs. RT induces DNA damage and ICD, leading to antigen release, dendritic cell activation and IFN-I signalling through the cGAS-STING pathway. These processes promote T-cell infiltration and antitumour immunity. ICIs counteract RT-induced PD-L1 upregulation and sustain cytotoxic T-cell activity, achieving durable systemic immune responses. Created in BioRender. Abbreviations: APCs: antigen-presenting cells; ATP: adenosine triphosphate; CAFs: cancer-associated fibroblasts; CCL22: chemokine (C-C motif) ligand 22; CRT: calreticulin; CTLs: cytotoxic T lymphocytes; cGAS: cyclic GMP-AMP synthase; cGAS-STING: cyclic GMP-AMP synthase–stimulator of interferon genes pathway; DAMPs: damage-associated molecular patterns; DCs: dendritic cells; ECs: endothelial cells; HSPs: heat shock proteins; HMGB1: high mobility group box 1; IL-10: interleukin-10; MDSCs: myeloid-derived suppressor cells; STING: stimulator of interferon genes; Tregs: regulatory T cells; TAMs: tumour-associated macrophages; TLRs: Toll-like receptors; TGF-β: transforming growth factor-β; IFN-I: type I interferons.

The mechanistic basis of the combination of RT and ICIs lies in the complementary antitumour mechanisms of the two modalities. While conventional RT has long been recognized for its efficacy in achieving local tumour control, it also induces ICD and promotes the release of tumour-associated antigens, thereby providing the initial molecular substrate for systemic immune activation. The synergistic mechanisms of the combination of RT and ICIs involve RT-induced DNA damage and activation of the cGAS-STING pathway, the initiation of antigen presentation through ICD and immunomodulation of the TME (Figure 1). Together, these processes enhance effector T cell infiltration, activation and memory formation, establishing a coherent mechanistic framework for the rational integration of RT with ICIs.

### Abscopal effect

The abscopal effect, first proposed by Mole in 1953, describes the regression of distant, non-irradiated tumour lesions following localized RT (Ref. 27). Long considered anecdotal, this phenomenon is now recognized as an immune-mediated process triggered by RT-induced tumour antigen release and systemic T cell activation (Ref. 55).

Mechanistically, tumour cell death induced by RT results in the release of neoantigens, which are captured by dendritic cells (DCs) and presented to naïve CD8^+^ T cells within tumour-draining lymph nodes (Ref. 15). Through this interaction, CD8^+^ T cells become activated and differentiate into cytotoxic T lymphocytes (CTLs) with tumour specificity. CTLs subsequently migrate to non-irradiated sites and mediate tumour cell apoptosis through perforin and granzyme B release (Ref. 56). In addition, studies have shown that p53 is a key tumour suppressor protein upregulated in response to radiation and plays an important role in RT-induced abscopal effects. Experimental data indicate that the absence of p53 significantly weakens the suppression of distant, non-irradiated tumours following radiation (Ref. 57).

Clinically, abscopal effects are rare with RT alone but occur more frequently when RT is combined with ICIs targeting PD-1/PD-L1 or CTLA-4 pathways (Refs 55, 58–61). This synergy reinforces the concept that localized irradiation can generate systemic immune activation, transforming RT into a ‘vaccine-like’ therapy with distant antitumour effects.

### ICD

ICD represents a cornerstone mechanism underlying the immune-sensitizing potential of RT. Unlike non-inflammatory apoptosis, ICD converts dying tumour cells into a source of immune stimulation by releasing damage-associated molecular patterns (DAMPs) such as high mobility group box 1 (HMGB1), calreticulin (CRT), adenosine triphosphate (ATP) and heat shock proteins (HSPs) (Refs 62–64). These molecules engage pattern recognition receptors (PRRs) on antigen-presenting cells, triggering DC maturation and antigen cross-presentation to T cells (Refs 65–69).

RT induces ICD primarily through ionizing radiation-mediated DNA damage, generation of reactive oxygen species (ROS), endoplasmic reticulum (ER) stress and mitochondrial injury (Ref. 70). Surface exposure of CRT acts as an ‘eat-me’ signal recognized by CD91 on DCs, while extracellular ATP activates the P2X7 receptor, promoting NLRP3 inflammasome activation and interleukin-1β (IL-1β) secretion (Refs 65, 66). Simultaneously, HMGB1 binds to Toll-like receptor 4 (TLR4), enhancing antigen presentation and T cell priming (Ref. 67).

Through these coordinated molecular events, RT-induced ICD transforms tumour cell death into an in situ vaccination process. When combined with ICIs, ICD enhances both the magnitude and duration of antitumour T cell responses, establishing durable systemic immunity and reducing recurrence risk (Refs 68, 69).

### The cGAS-STING pathway

The cGAS-STING pathway serves as a key molecular link between DNA damage caused by RT and systemic immune activation (Ref. 15). RT causes DNA DSBs in tumour cells through ionizing radiation, leading to the formation of chromosomal fragments or micronuclei (Refs 71, 72). Cytosolic accumulation of nuclear or mitochondrial DNA (mtDNA) fragments activates cyclic GMP-AMP synthase (cGAS), catalysing the formation of cyclic GMP-AMP (cGAMP), which binds to stimulator of interferon genes (STING) on the endoplasmic reticulum membrane. This interaction triggers downstream TBK1-IRF3-NF-κB signalling, leading to transcription of type I interferon (IFN-I) and proinflammatory cytokines (Refs 73–76).

IFN-I enhances DC maturation, migration to tumour-draining lymph nodes and activation of tumour-specific CD8^+^ T cells (Refs 77, 78). Preclinical studies have shown that an intact cGAS-STING axis in host DCs is essential for CD8^+^ T cell-dependent tumour regression after RT (Refs 71, 79).

Beyond nuclear DNA, RT also induces mitochondrial outer membrane permeabilization (MOMP), resulting in the release of mtDNA into the cytoplasm (Refs 80, 81). Because mtDNA is enriched in unmethylated CpG motifs and lacks histone protection, it may be more readily sensed by cGAS than nuclear DNA (Ref. 82). Radiation-induced mtDNA leakage has been demonstrated to activate cGAS-STING signalling and IFN-I production in breast cancer (BC), nasopharyngeal carcinoma (NPC) and colorectal adenocarcinoma (CA), thereby eliciting a robust antitumour immune response (Refs 83–86).

In addition to the mechanisms mentioned above, studies have found that irradiation of normal tissue surrounding the tumour has also been reported to trigger antitumour immune responses. This occurs even when the tumour itself is not directly exposed to radiation. The response has been shown to rely on the expression of p53 (Ref. 57). The underlying mechanism may involve p53 contributing to the cGAS-STING-mediated immune response by suppressing the three-prime repair exonuclease 1 (TREX1), thereby sustaining cytosolic DNA accumulation (Ref. 87).

Emerging evidence suggests that the cGAS-STING pathway functions as a context-dependent immune regulator with dual roles in antitumour immunity. Transient or moderate activation of cGAS-STING signalling promotes DC maturation and IFN-I production, thereby enhancing antigen presentation and priming adaptive immune responses (Refs 88, 89). At the same time, interferon-driven signalling can upregulate PD-L1 expression, providing a mechanistic basis for the synergy between RT and PD-1/PD-L1 blockade (Ref. 90).

In contrast, sustained or excessive activation of the cGAS-STING axis has been associated with progressive T cell dysfunction and the development of an exhausted phenotype, frequently in the context of prolonged IFN-I signalling and chronic inflammatory stimulation. Under such conditions, tumour-infiltrating T cells exhibit broader co-inhibitory receptor programmes, characterized by the concurrent expression of PD-1, LAG-3 and TIGIT, which collectively limit the efficacy of PD-1-based monotherapy (Refs 91, 92). Collectively, these observations indicate that the immunological outcome of cGAS-STING activation is critically shaped by both the magnitude and temporal dynamics of signalling, and provide a mechanistic rationale for evaluating next-generation immune checkpoint blockade, including anti-LAG-3 or anti-TIGIT strategies, in settings of adaptive or secondary immune resistance (Refs 47, 93, 94).

### TME remodelling

RT reshapes the TME by altering the abundance, phenotype and function of both immune and stromal cells. These modifications determine whether the irradiated milieu becomes immunostimulatory or immunosuppressive (Refs 95–98).

RT enhances the infiltration and activation of CTLs through increased antigen release, upregulation of MHC class I and induction of chemokines such as ICAM-1 and CXCL10 (Refs 71, 99–101). Activated CTLs produce perforin and granzyme B, mediate tumour cell killing and contribute to a systemic antitumour immune response (Ref. 56). However, recent studies show that RT-induced interferon signalling markedly upregulates tumour PD-L1, which, by engaging PD-1, dampens T cell activity and contributes to exhaustion; ICIs can reverse this effect, providing theoretical support for the combination (Ref. 102). Importantly, preclinical models indicate that RT-driven inflammatory remodelling is also associated with the emergence of alternative exhausted T cell states characterized by increased LAG-3 and TIGIT expression (Ref. 48). These findings suggest that RT may sensitize tumours to PD-1/PD-L1 blockade and simultaneously create a cellular context in which next-generation ICIs are required to fully restore T cell functionality.

Studies have shown that RT exerts a bidirectional effect on Tregs. RT exerts context dependent effects on Tregs, improving the effector-to-regulatory ratio, and checkpoint blockade further neutralizes their suppressive function (Ref. 103). In addition to its suppressive effects on Tregs, RT can induce the secretion of CCL22. Tregs, expressing the CCR4 receptor, bind to CCL22, which promotes their recruitment into the TME (Refs 104, 105). Tregs can suppress the proliferation of effector T cells (Teffs) and inhibit T cell activation by expressing CTLA-4 (Refs 106, 107). This immunosuppressive function provides a key rationale for the synergistic effect observed when RT is combined with ICIs. In addition, vascular damage caused by RT can worsen tumour hypoxia, leading to activation of the hypoxia-inducible factor 1-alpha (HIF-1α) pathway. This enhances FoxP3 expression and promotes the differentiation of CD4^+^ T cells into Tregs (Refs 108, 109).

RT-induced ICD releases DAMPs that promote DC maturation via PRR signalling. Mature DCs migrate to lymph nodes, upregulate costimulatory molecules and cross-present tumour antigens to T cells (Refs 110–112). The cGAS-STING pathway amplifies these effects by stimulating IFN-I secretion, which enhances DC activation and T cell priming. However, excessive radiation doses or oxidative stress can impair DC viability and function, underscoring the need for optimized RT fractionation (Ref. 88). ICIs may enhance the downstream efficacy of DC-mediated antigen presentation by sustaining T cell activation.

Tumour-associated macrophages (TAMs) are one of the major immunosuppressive components in the TME. RT can promote the release of cytokines such as TGF-β and IL-10, which facilitate the recruitment and polarization of TAMs (Refs 113–115). In addition, hypoxic conditions activate the HIF-1α signalling pathway, driving TAMs towards an immunosuppressive TAM phenotype (Ref. 116). These TAMs further express PD-L1 and secrete vascular endothelial growth factor (VEGF), enhancing immune evasion and abnormal angiogenesis (Refs 117–119). These findings further support the clinical rationale for combining RT with ICIs.

At the same time, RT-induced inflammatory responses can stimulate the release of MDSCs from the bone marrow. These cells are then recruited to and activated within the TME. RT can also temporarily recruit MDSCs through chemokines such as CCL2 and CXCL12 (Refs 11, 120). MDSCs suppress T cell activity by depleting amino acids through the enzyme arginase 1 (ARG1). Upon stimulation by RT, they may further enhance their immunosuppressive phenotype (Ref. 121). The extent and timing of MDSC modulation are key factors in achieving sustained immune activation.

Cancer-associated fibroblasts (CAFs) exhibit dual responses to radiation. Low-dose RT may suppress their activity and enhance immune infiltration, whereas higher doses activate fibroblasts and trigger secretion of TGF-β, IL-6 and IL-13, leading to fibrosis and T cell exclusion (Refs 122, 123). CAFs can also express inhibitory ligands such as CD253, CD252 and the immune checkpoint molecule CD276, directly dampening T cell activation (Ref. 122). Combining RT with ICIs or TGF-β blockade mitigates CAF-induced immunosuppression and facilitates T cell trafficking into the tumour parenchyma (Refs 124, 125).

RT modifies tumour vasculature by inducing endothelial apoptosis and upregulating adhesion molecules (Refs 126–128), thereby enhancing lymphocyte extravasation. However, radiation-induced hypoxia can activate HIF-1α and promote vascular remodelling towards an immunosuppressive phenotype (Refs 128, 129). ICIs may complement RT-induced vascular remodelling by sustaining antitumor T cell activity and facilitating lymphocyte infiltration within tumours.

## Clinical progress of RT combined with ICIs

**Figure 2.**
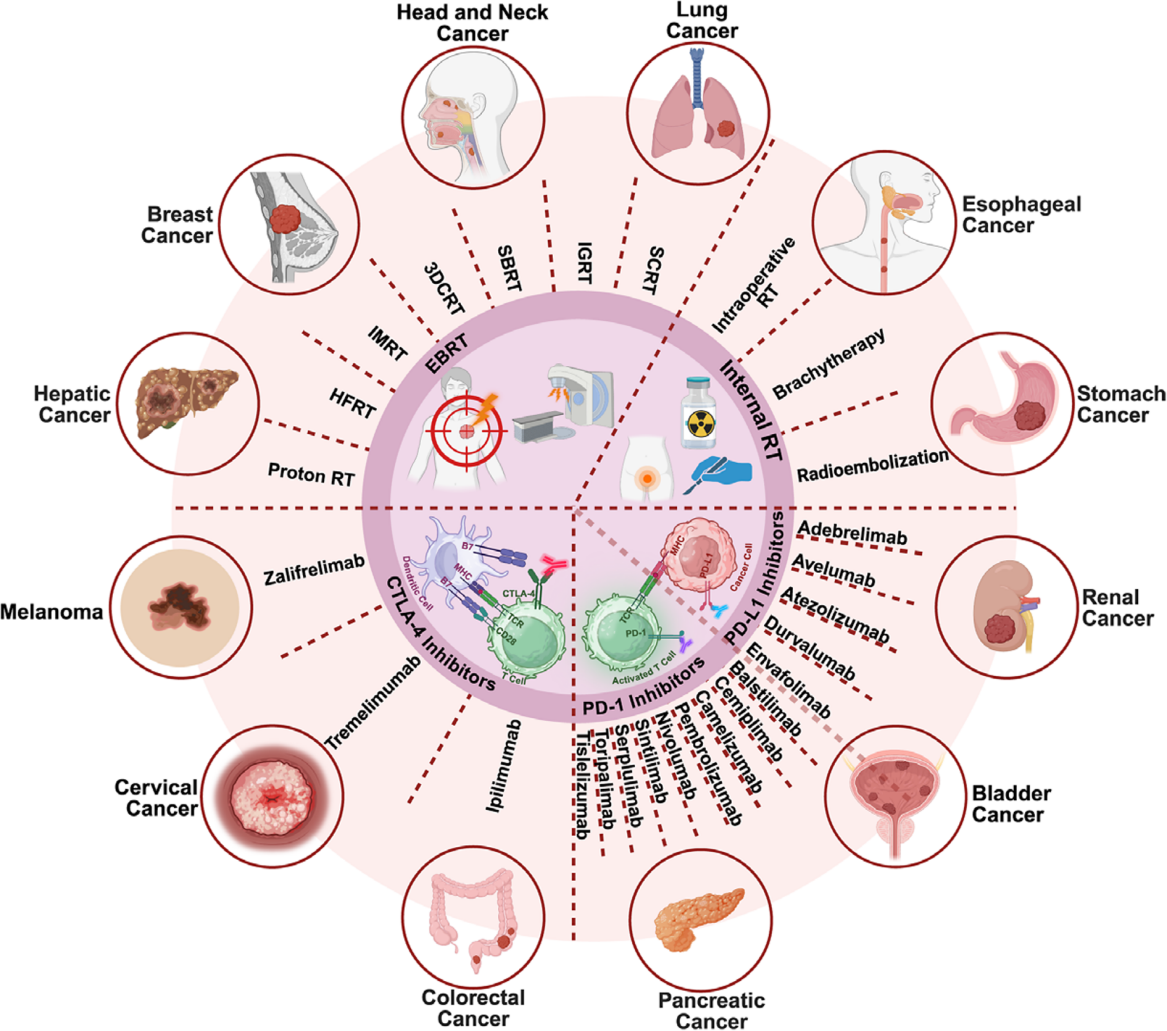
RT modalities, ICIs and their combinatorial applications in STs. Together, RT and ICIs reprogramme the TME towards an immune-active state, forming the mechanistic basis for emerging clinical combination strategies. Created in BioRender. Abbreviations: CTLA-4: cytotoxic T lymphocyte antigen 4; EBRT: external beam radiation therapy; HFRT: hypofractionated radiation therapy; IGRT: image-guided radiotherapy; IMRT: intensity-modulated radiation therapy; PD-1: programmed cell death 1; PD-L1: programmed cell death ligand 1; SBRT: stereotactic body radiation therapy; SCRT: short-course radiotherapy; 3DCRT: three-dimensional conformal radiation.

The integration of RT and ICIs has demonstrated encouraging efficacy across multiple STs. Mechanistically, ICIs enhance cytotoxic T cell activity by blocking inhibitory pathways such as PD-1, PD-L1 and CTLA-4, while RT augments tumour immunogenicity through antigen release, vascular normalization and immune infiltration. Their combination has shown synergistic potential to improve local control, reduce metastasis and prolong survival (Refs 42, 130–132). However, clinical outcomes vary widely due to heterogeneity in RT modalities, dose fractionation and ICIs scheduling. Ongoing trials aim to define optimal regimens, biomarkers and patient selection strategies to maximize benefit and minimize toxicity (Figure 2).

### PD-1 inhibitors

PD-1 inhibitors – including Camrelizumab, Nivolumab, Pembrolizumab, Toripalimab, Tislelizumab, Sintilimab and Serplulimab – have shown consistent synergy with RT across a wide spectrum of malignancies (Table 1).

**Table 1.** Completed clinical studies on RT combined with PD-1 inhibitors (in the past 3 years)

Disease	Trial registration	Phase	ICIs		RT			Other treatments	Primary endpoint	Results	References
			Type	Dose	Type	Total dose	Fraction dose				
STs	ChiCTR1900026175	Phase 2	PD–1 inhibitors	/	SBRT, HFRT	15–24Gy	5–8Gy	GM-CSF	ORR	ORR 16.7%	Ref. 133
ESCC	ChiCTR2000040533	Phase 2	Camrelizumab	200 mg, q3w, IV	RT	30–40Gy	3–2Gy	Irinotecan	PFS	Median PFS 6.9 months	Ref. 134
GC	NCT03631615	Phase 2	Camrelizumab	200 mg, q3w, IV	IMRT	45Gy	1.8Gy	Oxaliplatin, Capecitabine, Surgery	pCR rate	pCR 33.3%	Ref. 135
HCC	NCT04193696	Phase 2	Camrelizumab	200 mg, q3w, IV	SBRT	20–30– 40Gy	2–3–4Gy	/	ORR	ORR 52.4%	Ref. 136
HNSCC	NCT04156698	Phase 2	Camrelizumab	200 mg, q3w, IV	RT	66–70Gy	2.2–2Gy	Capecitabine, Cisplatin, Docetaxel	ORR	ORR 82.4%	Ref. 137
iCCA	NCT03898895	Phase 2	Camrelizumab	200 mg, q3w, IV	IMRT	45Gy	2–2.5Gy	/	PFS	PFS 44.4%	Ref. 138
Metastatic STs	ChiCTR2200062706	Phase 2	Camrelizumab	200 mg, q3w, IV	HFRT	24–56Gy	4–10Gy	GM-CSF, Thymosin-α1	Median PFS	Median PFS 3.5 months	Ref. 139
NPC	ChiCTR2000032317	Phase 2	Camrelizumab	200 mg, q3w, IV	IMRT	54–72Gy	1.8–2.4Gy	Apatinib, Capecitabine, Cisplatin, Paclitaxel	DMFS	DMFS 98%	Ref. 140
RC	NCT04340401	Phase 2	Camrelizumab	200 mg, IV	IMRT	50.4–54Gy	2.3–2.5Gy	Capecitabine, Oxaliplatin, Surgery	pCR rate	pCR rate 33.3%	Ref. 141
STs	NCT04569916	Phase 2	Camrelizumab	200 mg, q3w, IV	RT	15–24Gy	5–8Gy	Apatinib, Irinotecan liposome	ORR	ORR 34.6%	Ref. 142
CC	NCT03298893	Phase 1	Nivolumab	240 mg, q2w, IV	RT	45–54Gy	1.8–2.16Gy	Cisplatin	DLT	DLT 20% (n = 15)	Ref. 143
				1 mg/kg, q2w, IV							
HCC	NCT03380130	Phase 2	Nivolumab	240 mg, q2w, IV	Radioembolization	120Gy	/	/	Safety	Safe	Ref. 144
						40Gy	/				
NPC	NCT03984357	Phase 2	Nivolumab	360 mg, q3w	IMRT	60.96Gy	2.12Gy	Gemcitabine, Cisplatin	FFS	FFS 88.5%	Ref. 145
				480 mg, q4w							
NSCLC	NCT03110978	Phase 2	Nivolumab	480 mg, q4w, IV	SBRT	50–60–70– 80Gy	12.5–15– 7–8Gy	/	EFS	SBRT EFS: 53%	Ref. 146
				240 mg, q2w, IV						SBRT + Nivolumab EFS: 77%	
GBM	NCT02617589	Phase 3	Nivolumab	280 mg, q2w	RT	60Gy	2Gy	Temozolomide	OS	The study did not meet the primary endpoint of improved OS	Ref. 16
HCC	NCT03099564	Phase 1	Pembrolizumab	200 mg, q3w, IV	Radioembolization	/	/	/	PFS	PFS 55.6%	Ref. 17
STs	NCT02608385	Phase 1	Pembrolizumab	200 mg, q3w, IV	SBRT	30–50Gy	10Gy	/	Safety	Safe	Ref. 147
RCC	NCT02855203	Phase 1 Phase 2	Pembrolizumab	200 mg, q3w, IV	SBRT	18–20Gy	18–20Gy	/	Safety	Safe	Ref. 148
MIBC	NCT02662062	Phase 2	Pembrolizumab	200 mg, q3w, IV	IMRT	64Gy	2Gy	Cisplatin	Feasibility	Feasible	Ref. 149
					3DCRT	64Gy	2Gy				
NSCLC	NCT03087760	Phase 2	Pembrolizumab	200 mg, q3w, IV	Proton RT	60–70Gy	1.8–2.0– 4.0Gy	Platinum-based doublet chemotherapy	PFS	Median PFS 8.8 months	Ref. 150
PC	NCT02704156	Phase 2	Pembrolizumab	200 mg, q3w, IV	SBRT	35–40Gy	7–8Gy	Trametinib, Gemcitabine	OS	SBRT + Pembrolizumab + Trametinib Median OS: 14.9 months SBRT + Gemcitabine Median OS: 12.8 months	Ref. 151
TNBC	NCT03004183	Phase 2	Pembrolizumab	200 mg, q3w, IV	SBRT	30Gy	6Gy	ADV/HSV-tk, Valacyclovir	CBR	CBR 21.4%	Ref. 152
CC	NCT04221945	Phase 3	Pembrolizumab	200 mg, q3w, IV	EBRT	80Gy	2Gy	Cisplatin	PFS	Median PFS was not reached	Ref. 153
				400 mg, q6w, IV	Brachytherapy	75Gy	2Gy		OS	Experimental cohort: OS 87% Observation cohort: OS 81%	
HNSCC	NCT03040999	Phase 3	Pembrolizumab	200 mg, q3w, IV	RT	70Gy	2Gy	Cisplatin	EFS	No difference	Ref. 154
STs	NCT05501340	Phase 1 Phase 2	Serplulimab	100 mg, IP	HFRT, SBRT	24Gy	8Gy	Molgramostim	Safety (Phase 1)	Safe	Ref. 155
				3 mg/kg, q2w, IV					OS (Phase 2)	OS data need to be verified	
ESCC	ChiCTR1900027161	Phase 1b Phase 2	Sintilimab	200 mg, q3w, IV	RT	45–50.4Gy	1.8Gy	Raltitrexed	Safety	Safe	Ref. 156
									OS	Median OS 20.6 months, OS rate 64%	
CC	ChiCTR2000032879	Phase 1 Phase 2	Toripalimab	240 mg, q3w, IV	EBRT	45–50.4Gy	1.8Gy	Cisplatin	Safety	Safe	Ref. 157
					Brachytherapy	24–30Gy	8–6Gy		PSF	PSF 81.8%	
						80–90Gy	2Gy				
ESCC	ChiCTR2100046715	Phase 2	Toripalimab	240 mg, q3w, IV	RT	50–50.4Gy	2–1.8Gy	Paclitaxel, Carboplatin	PFS	Despite the primary endpoint was not met	Ref. 158
					SBRT	30–40Gy	10–8Gy		ORR		
RC	NCT04518280	Phase 2	Toripalimab	240 mg, q3w, IV	SCRT	25Gy	5Gy	Capecitabine, Oxaliplatin, Surgery	CR rate	Experimental group CR rate: 56.5% Control group CR rate: 54.2%	Ref. 159
ESCC	ChiCTR2100054327	Phase 2	Tislelizumab	200 mg, q3w, IV	RT	50Gy	2Gy	Cisplatin, Paclitaxel	PFS	PFS 79.4%	Ref. 160
NSCLC	NCT05319574	Phase 2	Tislelizumab	200 mg, q3w, IV	SBRT	24Gy	8Gy	Surgery, Platinum-based doublet chemotherapy	MPR	MPR 76%	Ref. 161
HCC	ChiCTR2000036385	Phase 2	Tislelizumab	200 mg, q3w, IV	IMRT	45Gy	3Gy	/	ORR	ORR 30%	Ref. 162
									OS	Median OS 18.7 months	

Abbreviations: 1. CC: cervical cancer; ESCC: oesophageal squamous cell cancer; GC: gastric cancer; GBM: glioblastoma; HCC: hepatocellular cancer; HNSCC: head and neck squamous cell cancer; iCCA: intrahepatic cholangiocarcinoma; MIBC: muscle invasive bladder cancer; NPC: nasopharyngeal cancer; NSCLC: non-small cell lung cancer; PC: pancreatic cancer; RC: rectal cancer; RCC: renal cell cancer; STs: solid tumours; TNBC: triple negative breast cancer.

2. IV: intravenous injection.

3. EBRT: external beam radiotherapy; HFRT: hypofractionated radiation therapy; IMRT: intensity modulated radiation therapy; RT: radiation therapy; SBRT: stereotactic body radiation therapy; SCRT: short-course radiotherapy; 3DCRT: three-dimensional conformal external beam radiation.

4. ADV/HSV-tk: adenovirus-mediated expression of herpes-simplex-virus thymidine kinase; GM-CSF: granulocyte macrophage–colony stimulating factor.

5. CBR: clinical benefit rate; CR: complete response; DLT: dose-limiting toxicity; DMFS: distant metastasis-free survival; EFS: event-free survival; FFS: failure-free survival; MPR: major pathological response; ORR: objective response rate; OS: overall survival; PFS: progression-free survival; pCR: pathological complete response.

Camrelizumab is a humanized IgG4 anti-PD-1 monoclonal antibody that has been approved for the treatment of cutaneous squamous cell carcinoma (cuSCC). It has also shown promising potential across various STs. Its synergy with RT arises from the interplay of multiple immune-related mechanisms (Refs 134, 142). In recent years, clinical studies investigating the combination of Camrelizumab with RT in the treatment of various STs have been steadily advancing. These studies have demonstrated promising efficacy and favourable safety profiles. The study by Zhao et al. demonstrated that in patients with ESCC who had failed prior chemotherapy, the combination of Camrelizumab with low-dose RT and irinotecan as second-line treatment significantly prolonged progression-free survival (PFS) and overall survival (OS). Moreover, the treatment showed a clear synergistic effect along with manageable safety, indicating its potential as an effective and tolerable therapeutic option (Ref. 134). In neoadjuvant treatment for gastric adenocarcinoma, Camrelizumab combined with concurrent chemoradiotherapy (CCRT) achieved a pathological complete response (pCR) rate of 33.3%. This highlights the role of RT-induced immune enhancement in improving the efficacy of ICIs (Ref. 139). In hepatocellular cancer (HCC), the combination of Camrelizumab with SBRT achieved an objective response rate of 52.4%, which is superior to historical data for monotherapy. This finding further supports the hypothesis that RT can enhance the efficacy of ICIs by inducing a potent antitumour immune response (Ref. 136). Similar outcomes have been observed in other types of STs. In patients with hypopharyngeal squamous cell carcinoma, the combination of Camrelizumab with induction chemoradiotherapy (CRT) achieved an objective response rate of 82.4% (Ref. 137). In NPC, Camrelizumab combined with CRT and the anti-angiogenic agent apatinib resulted in a 1-year distant metastasis-free survival rate of 98% (Ref. 140). Additionally, in unresectable intrahepatic cholangiocarcinoma (iCCA), Camrelizumab combined with RT as a first-line treatment yielded an objective response rate of 61.1%, with manageable toxicity (Ref. 138). In addition to the above findings, studies exploring the combination of Camrelizumab with RT and irinotecan liposome in the treatment of advanced STs have also demonstrated significant clinical benefits. These results further underscore the important role of Camrelizumab in expanding therapeutic options for advanced solid malignancies (Ref. 142). Collectively, available studies suggest that Camrelizumab, particularly when combined with RT and other systemic treatments, exhibits significant synergistic effects and is emerging as a key component of multimodal cancer therapy strategies. Ongoing clinical trials are actively evaluating Camrelizumab in combination with RT across multiple tumour types (Supplementary Table 1).

Nivolumab is also a widely used human monoclonal antibody targeting PD-1. It is now widely used in the treatment of various STs, including NSCLC, HNSCC, RCC and HCC. In recent years, the combination of Nivolumab with RT has demonstrated significant synergistic effects in various STs, establishing itself as an important component of multimodal antitumour strategies. In the treatment of cervical cancer (CC), Nivolumab combined with CCRT has demonstrated an objective response rate (ORR) of up to 93.8% and significantly prolonged PFS. These results suggest a substantial advantage in enhancing immune-mediated tumour control (Ref. 143). In patients with HCC, the combination of Nivolumab with selective internal radiotherapy (SIRT) achieved an ORR of 41.5%, which is markedly superior to previous monotherapy outcomes. This suggests that the combination effectively activates antitumour immune pathways and enhances the therapeutic efficacy of RT (Ref. 144). In the treatment of NPC, the combination of Nivolumab with induction chemotherapy and RT has also shown excellent efficacy. It significantly improved failure-free survival (FFS), with a 3-year FFS rate of 88.5%. Notably, this regimen eliminated the need for concurrent cisplatin, thereby reducing the toxicity associated with traditional chemotherapy (Ref. 145). In the treatment of NSCLC, perioperative administration of Nivolumab combined with chemotherapy in stage III patients has significantly improved pCR rates and OS. These findings confirm its clinical value in the neoadjuvant setting (Ref. 163). In addition, the combination of Nivolumab with SBRT significantly improved 4-year event-free survival (EFS), increasing it from 53% to 77%. This further supports its potential to activate systemic immune responses and extend long-term survival (Ref. 146). Although Nivolumab has shown significant efficacy in combination therapies across various STs, its therapeutic outcomes remain somewhat tumour type dependent. In glioblastoma, the combination of Nivolumab with RT did not demonstrate superiority over temozolomide, suggesting that the complex TME may limit its effectiveness. This underscores the importance of tumour-specific immunotherapeutic strategies and precise patient selection (Ref. 16).

Following Nivolumab, another widely used PD-1 inhibitor in the ICIs of STs is Pembrolizumab. In the treatment of locally advanced CC, Pembrolizumab combined with CRT has significantly prolonged PFS, reinforcing its added benefit within standard treatment protocols (Ref. 153). In the treatment of recurrent NSCLC, consolidation therapy with Pembrolizumab following thoracic proton reirradiation has also shown favourable outcomes, with a median PFS of 8.8 months. This highlights the clinical value of integrating precision RT with ICIs (Ref. 150). In the treatment of HCC, Pembrolizumab combined with yttrium-90 (Y90) radioembolization has demonstrated durable antitumour activity, with a PFS of approximately 10 months and OS exceeding 2 years. This combination is particularly beneficial for patients with poor prognosis and difficult-to-treat HCC (Ref. 17). In patients with oligometastatic RCC, short-course Pembrolizumab administered after SBRT has achieved excellent local control and durable responses (Ref. 148). In muscle-invasive bladder cancer (MIBC), the combination of Pembrolizumab with CRT has achieved a favourable 2-year complete response (CR) rate, along with effective control of distant metastases. These results provide strong support for bladder-preserving multimodal treatment strategies (Ref. 149). In addition, in patients with postoperative, locally recurrent pancreatic cancer (PC), the combination of Pembrolizumab and trametinib with SBRT significantly improved OS. Compared to conventional chemotherapy regimens, this approach demonstrated superior therapeutic benefit, highlighting the strong synergistic potential of combining ICIs, targeted therapy and RT (Ref. 151). Moreover, in the treatment of advanced STs, Pembrolizumab combined with SBRT has demonstrated favourable safety and efficacy. Notably, in patients receiving SBRT to only a subset of lesions, OS was significantly improved. This highlights the regulatory potential of SBRT in stimulating systemic immune responses (Ref. 147). It is worth noting that in studies on metastatic TNBC, the combination of Pembrolizumab with stereotactic RT and oncolytic gene therapy has been explored. However, the clinical benefits observed were relatively limited, suggesting that challenges such as insufficient immunogenicity and immune tolerance may still hinder effectiveness in certain tumour types (Ref. 152).

Sintilimab, Serplulimab, Toripalimab and Tislelizumab are next-generation PD-1 inhibitors independently developed in China. In recent years, clinical studies involving their combination with other therapies across various STs have been actively advancing (Table 1), demonstrating promising efficacy and favourable safety profiles. In ESCC, Sintilimab used as consolidation therapy following CCRT significantly extended OS to 20.6 months. It showed a favourable safety profile and durable responses, highlighting its potential value for further investigation in larger-scale clinical studies (Ref. 156). In the ‘PRaG’ regimen, Serplulimab combined with hypofractionated radiotherapy (HFRT) and GM-CSF has shown promising therapeutic potential in patients with advanced, treatment-refractory ST accompanied by malignant ascites. This approach highlights the innovative value of intraperitoneal drug delivery strategies in complex clinical settings (Ref. 155). Toripalimab, when combined with definitive CRT in cervical squamous cell cancer (CSCC), has demonstrated excellent efficacy. The two-year PFS rate reached 81.8%, and the local control rate was as high as 95.5%, underscoring its potential as a powerful complement to conventional CRT (Ref. 157). In addition, the combination of Toripalimab with chemotherapy and RT in the treatment of advanced ESCC has also resulted in high response rates and notable survival benefits (Ref. 158). Tislelizumab has also shown great promise in various perioperative RT combination strategies. In unresectable locally advanced ESCC, Tislelizumab combined with CRT as a conversion therapy significantly improved tumour resectability and OS, with a satisfactory pathological response rate (Ref. 160). In resectable NSCLC, the combination of Tislelizumab with SBRT followed by chemotherapy achieved a major pathological response (MPR) rate of up to 76%. This demonstrates strong potential for improving postoperative outcomes (Ref. 161). In HCC with major vascular invasion, Tislelizumab combined with IMRT as perioperative treatment has also yielded significant response and survival benefits. This supports its therapeutic value in the management of aggressive STs (Ref. 162).

Overall, the multimodal combination strategies of PD-1 inhibitors with RT have demonstrated consistent synergistic efficacy and manageable toxicity across multiple tumour types. However, treatment outcomes and safety are still influenced by factors such as tumour type, stage and therapeutic context. Future efforts should focus on large-scale randomized controlled trials and biomarker-driven personalized approaches to further optimize these regimens, enhance therapeutic efficacy and minimize adverse effects. These combination strategies hold the potential to redefine treatment paradigms in STs and advance the development of precision radioimmunotherapy.

### PD-L1 inhibitors

Beyond PD-1 blockade, PD-L1 inhibitors – including Atezolizumab, Durvalumab, Avelumab and Adebrelimab – have demonstrated clinical benefit in combination with RT (Table 2).

**Table 2.** Completed clinical studies on RT combined with PD-L1 inhibitors (in the past 3 years)

Disease	Trial registration	Phase	ICIs		RT			Other treatments	Primary endpoint	Results	References
			Type	Dose	Type	Total dose	Fraction dose				
TNBC	NCT05132790	A pilot study	Adebrelimab	20 mg/kg, q3w, IV	SBRT	24Gy	8Gy	Paclitaxel, Carboplatin, Surgery	pCR rate	pCR 90%	Ref. 164
NSCLC	NCT03644823	Phase 2	Atezolizumab	1,200 mg, q3w, IV	SBRT	18Gy	6Gy	/	Toxicity	Grade 3 toxicity: 3 patients (n = 21)	Ref. 165
NSCLC	NCT02525757	Phase 2	Atezolizumab	1,200 mg, q3w, IV	RT	60–66Gy	2Gy	Carboplatin, Paclitaxel	Safety	Safe	Ref. 166
NSCLC	NCT03102242	Phase 2	Atezolizumab	1,200 mg, q3w, IV	RT	60Gy	2Gy	Carboplatin, Paclitaxel	DCR	DCR 74.2%	Ref. 167
SCLC	NCT04622228	Phase 2	Atezolizumab	1,200 mg, q3w, IV	Thoracic RT	15Gy	3Gy	Platinum-based chemotherapy, Etoposide	ORR	ORR 73.3%	Ref. 168
GM	NCT03217747	Phase 1 Phase 2	Avelumab	10 mg/kg, q2w, IV	RT	60Gy	6Gy	Utomilumab, PF-04518600	Safety	Safe	Ref. 169
HCC	NCT03817736	Phase 2	Avelumab	10 mg/kg, q2w, IV	SBRT	27.5Gy– 40Gy	5.5–8Gy	TACE	Proportion of patients deemed amenable to curative treatment	18 (55%) patients were deemed amenable to curative treatment	Ref. 170
STs	NCT03724890	Phase 1	Avelumab	800 mg, q2w, IV	RT	30Gy	3Gy	Peposertib	DLT	Peposertib + avelumab DLT: Peposertib 250 mg 1 patient, 300 mg 1 patient, 400 mg 4 patients	Ref. 171
										Peposertib + avelumab + RT DLT: No	
NSCLC	NCT03693300	Phase 2	Durvalumab	1,500 mg, q4w, IV	RT	60Gy	/	Platinum-based chemotherapy	TRAEs	Incidence of Grade 3 or 4 TRAEs: 18.8% (n = 117)	Ref. 172
NSCLC	NCT03141359	Phase 2	Durvalumab	10 mg/kg, q4w, IV	SBRT	50–54Gy	10–18Gy	Carboplatin, Paclitaxel, Cisplatin, Etoposide	PFS	PFS 62.7%	Ref. 173
				1,500 mg, q4w, IV	RT	60Gy	2Gy				
HNSCC	NCT03051906	Phase 1 Phase 2	Durvalumab	1,500 mg, q4w, IV	IMRT	69.9Gy	2.12Gy	Cetuximab	PFS	PFS 58.3%	Ref. 174
NMIBC	NCT03317158	Phase 1	Durvalumab	1,120 mg, q3w, IV	EBRT	18Gy	6Gy	BCG	RP2D	Full-dose D, full-dose BCG and 6 Gy fractions 3 were determined as the RP2Ds	Ref. 175
RC	NCT04083365	Phase 2	Durvalumab	1,500 mg, q4w, IV	RT	50.4Gy	1.8– 2.0Gy	Capecitabine, Surgery	pCR rate	pCR rate 34.5%	Ref. 176
HCC	NCT04124991	Phase 1 Phase 2	Durvalumab	1,500 mg, q4w, IV	Radioembolization	100–150Gy	/	/	To evaluate TTP	Median TTP 15.2 months	Ref. 177
						400–800Gy	/				

Abbreviations: 1. HCC: hepatocellular cancer; HNSCC: head and neck squamous cell cancer; GM: gynaecologic malignancies; NSCLC: non-small cell lung cancer; NMIBC: non-muscle-invasive bladder cancer; RC: rectal cancer; SCLC: small cell lung cancer; STs: solid tumours; TNBC: triple negative breast cancer.

2. IV: intravenous injection.

3. EBRT: external beam radiation therapy; IMRT: intensity modulated radiation therapy; RT: radiation therapy; SBRT: stereotactic body radiation therapy.

4. BCG: bacillus calmette guerin; TACE: transarterial chemoembolization.

5. DCR: disease control rate; DLT: dose limiting toxicity; ORR: objective response rate; pCR: pathological complete response; PFS: progression-free survival; RP2D: recommended phase 2 dose; TTP: time to progression; TRAEs: treatment-related adverse events.

Adebrelimab is a novel high-affinity PD-L1 monoclonal antibody that has shown promising results in early clinical evaluations when combined with RT. In a prospective, single-arm phase 2 study, Chen et al. explored the feasibility of combining Adebrelimab with SBRT and platinum-based chemotherapy as neoadjuvant therapy for patients with TNBC. Among the 10 patients who underwent surgery, 90% achieved a pCR. Notably, no significant RT-related toxicity was observed (Ref. 164). These results suggest that Adebrelimab may enhance the immunogenic effects of RT and serve as an effective radiosensitizer in early-stage TNBC patients.

Atezolizumab, the first approved PD-L1 monoclonal antibody, has been widely applied across various tumour types, with the most extensive research conducted in the field of lung cancer. In the context of RT combination strategies, atezolizumab has also been one of the most extensively studied agents. In a clinical study by Ross et al., a three-phase treatment approach – comprising immune induction, CCRT and immune maintenance – was proposed for patients with unresectable stage III NSCLC. The results demonstrated a high disease control rate and substantial survival benefit, with a 24-month overall survival rate reaching 73.7% (Ref. 167). However, the incidence of grade ≥ 3 immune-related adverse events (irAEs) reached 27.4%, indicating the need for further evaluation of its safety and the identification of appropriate patient populations in larger-scale studies (Ref. 167). In extensive-stage small cell lung cancer (ES-SCLC), Wang et al. evaluated the feasibility of atezolizumab combined with low-dose RT as a first-line treatment. The results showed a notable increase in the ORR to 87.5% and a deepened immune response. The proposed mechanism is that low-dose RT facilitates the recruitment and activation of Tcf1-expressing stem-like CD8^+^ T cells, thereby converting immunologically ‘cold’ tumours into ‘hot’ tumours and enhancing systemic antitumour immune responses (Ref. 168).

Avelumab, when combined with RT, has shown significant potential in conversion therapy for locally advanced tumours, such as HCC. A prospective phase II clinical trial was the first to evaluate the efficacy and safety of a triple combination strategy – transarterial chemoembolization (TACE), SBRT and avelumab – in patients with unresectable HCC. The study results showed that among 33 patients, 55% achieved treatment conversion and 42% achieved radiological CR. Some patients subsequently underwent curative resection or ablation, demonstrating substantial clinical potential for this combination strategy. The incidence of irAEs of grade ≥ 3 was 15%, primarily involving immune-mediated hepatitis and dermatologic toxicity. Overall, the regimen was well tolerated, indicating its potential as an effective approach for conversion therapy (Ref. 170). In addition, a phase I study investigating Avelumab in combination with the DNA-PK inhibitor Peposertib, with or without RT, in patients with STs yielded disappointing results. Although the combination is mechanistically sound – aiming to enhance immune activation through DNA damage pathways – it did not show clear efficacy in an unselected patient population. This suggests that such strategies may require biomarker-driven patient selection, particularly based on DDR deficiencies or STING pathway activity, to optimize therapeutic benefit (Ref. 171).

Following the promising outcomes of atezolizumab and avelumab combined with RT across various tumour types, another extensively studied PD-L1 inhibitor – Durvalumab – has also been actively investigated in multiple malignant STs. Research on the synergistic effects of Durvalumab with RT continues to grow, further expanding the therapeutic landscape of PD-L1 monoclonal antibodies in combined radioimmunotherapy strategies. In HCC, Lee et al. conducted a clinical study using Y90 radioembolization combined with Durvalumab in patients with unresectable, locally advanced HCC. The results showed an impressive ORR of 83.3%, with 29.2% of patients achieving radiological CR. No severe toxicities were observed, indicating that this combination offers both high efficacy and a favourable safety profile (Ref. 177). In locally advanced rectal cancer, a phase II study evaluated a treatment strategy involving sequential administration of Durvalumab following long-course CRT. The results showed a pCR rate of 34.5%, indicating that this combination may enhance the quality of neoadjuvant therapy and provide a stronger foundation for effective tumour control prior to surgery (Ref. 176). In HNSCC, a phase 1/2 clinical trial investigated a triple combination therapy involving Durvalumab, cetuximab and IMRT. Although the trial was prematurely terminated due to slow patient accrual and included only 9 participants, follow-up data revealed 1-year and 2-year PFS rates of 77.7% and 58.3%, respectively. These findings suggest promising preliminary antitumour activity and tolerability, warranting further validation in larger-scale studies (Ref. 174). In the context of bladder cancer, a phase I multicentre study evaluated the safety and preliminary efficacy of Durvalumab in combination with Bacillus Calmette-Guérin (BCG) or EBRT in patients with non-muscle-invasive bladder cancer (NMIBC) who had failed prior BCG therapy. In the Durvalumab plus EBRT group, the CR rates at 3 and 12 months were 50% and 33%, respectively. The regimen was generally well tolerated, with only one case of grade 3 immune-related hepatitis reported. These findings suggest that this combinatorial approach may offer a promising local control strategy for patients unresponsive to conventional ICIs (Ref. 175).

In summary, the combination of Durvalumab and RT has demonstrated notable clinical activity and manageable toxicity across various STs, indicating its promising feasibility. However, large-scale randomized controlled trials are needed to further validate its efficacy. Future efforts should also focus on integrating molecular biomarker-driven patient selection strategies to optimize treatment populations, enhance response rates and improve survival outcomes.

### Dual ICIs

Dual ICIs – most commonly PD-1/PD-L1 plus CTLA-4 inhibition – represent an intensified approach to overcome immune resistance. When coupled with RT, this strategy has yielded enhanced responses in several STs (Table 3).

**Table 3.** Completed clinical studies on RT combined with multiple ICIs (in the past 3 years)

Disease	Trial registration	Phase	ICIs		RT			Other treatments	Primary endpoint	Results	References
			Type	Dose	Type	Total dose	Fraction dose				
HCC	NCT03203304	Phase 1	Nivolumab	240 mg, q2w, IV	SBRT	40Gy	8Gy	/	DLT	Nivolumab + Ipilimumab cohort DLT 14.3% Nivolumab cohort DLT 16.7%	Ref. 178
			Ipilimumab	1 mg/kg, q6w, IV							
HNSCC	NCT03162731	Phase 1	Nivolumab	3 mg/kg, q2w, IV	RT	70Gy	2Gy	/	TRAEs	Incidence of grade 3 or higher TRAEs: 88% (n = 24)	Ref. 179
			Ipilimumab	1 mg/kg, q6w, IV							
NSCLC	NCT02696993	Phase 1 Phase 2	Nivolumab	3 mg/kg, q2w, IV	SBRT	/	/	/	Safety	Safe	Ref. 180
				480 mg, q4w, IV							
			Ipilimumab	1 mg/kg, q6w, IV	Whole-Brain RT	16–18–20Gy	16–18– 20Gy	Intracranial PFS	Intracranial PFS 79.7%		
NSCLC	NCT03223155	Phase 1	Nivolumab	3 mg/kg, q2w, IV	SBRT	30–45–50Gy	10–15– 10Gy	/	DLT	Concurrent cohort: DLT rate of 0%	Ref. 181
			Ipilimumab	1 mg/kg, q6w, IV						Sequential cohort: DLT rate of 50%	
NSCLC	NCT04245514	Phase 2	Nivolumab	360 mg,	RT	50–60Gy	2Gy	Platinum-based chemotherapy, Surgery	Safety	Safe	Ref. 182
			Ipilimumab	1 mg/kg,					pCR	pCR 58%	
PC	NCT04258150	Phase 2	Nivolumab	1 mg/kg, q6w, IV	SBRT	15Gy	/	Tocilizumab	ORR	No objective responses observed	Ref. 183
			Ipilimumab	6 mg/kg, q4w, IV							
SCLC	NCT02046733	Phase 2	Nivolumab	1 mg/kg, q3w, IV	Thoracic RT	45–56Gy	1.5–5Gy	Platinum-based chemotherapy, Etoposide	PFS	Experimental cohort: PFS 43.2%	Ref. 184
				240 mg, q2w, IV	PCI	25 Gy	2.5Gy			Observation cohort: PFS 40.3%	
			Ipilimumab	3 mg, q3w, IV							
HNSCC	NCT03450967	Phase 2	Durvalumab	1,500 mg, q4w, IV	PT	25Gy	5Gy	/	ORR	ORR 22.6% (n = 31)	Ref. 185
			Tremelimumab	75 mg, q4w, IV							
HNSCC	NCT03426657	Phase 2	Durvalumab	1,500 mg, q4w, IV	RT	70–63–54Gy	2.0–1.8– 1.6Gy	Cisplatin, Docetaxel	Feasibility	Feasibility rate of 82% (n = 56)	Ref. 186
			Tremelimumab	75 mg, q4w, IV							
NSCLC	NCT02888743	Phase 2	Durvalumab	1,500 mg, q4w, IV	Low-dose RT	8Gy	0.5Gy	/	ORR	No difference	Ref. 187
			Tremelimumab	75 mg, q4w, IV	HFRT	24Gy	8Gy				
NSCLC	NCT03176173	Phase 2	Atezolizumab	/	IGRT	27–40–50Gy	9–4–5– 10Gy	Pemetrexed	PFS	PFS 60%	Ref. 188
			Nivolumab								
			Pembrolizumab								
STs	NCT04571632	Phase 2	Avelumab	40 mg, q2w, IT	SBRT	24Gy	8Gy	myDCs	PFS	Experimental Group PFS: 10%	Ref. 189
			Ipilimumab	10 mg, q2w, IT							
			Pembrolizumab	200 mg, q3w, IV						Control group PFS: 0%	

Abbreviations: 1. HCC: hepatocellular cancer; HNSCC: head and neck squamous cell cancer; NSCLC: non-small cell lung cancer; PC: pancreatic cancer; SCLC: small cell lung cancer; STs: solid tumour.

2. IT: intratumoural injection; IV: intravenous injection.

3. PCI: prophylactic cranial irradiation; PT: proton therapy; HFRT: hypofractionated radiation therapy; IGRT: image-guided RT; SBRT: stereotactic body radiation therapy; RT: radiation therapy.

4. DLT: dose limiting toxicity; ORR: objective response rate; PFS: progression free survival; TRAEs: treatment-related adverse events; pCR: pathological complete response.

The combination of Nivolumab (a PD-1 inhibitor) and Ipilimumab (a CTLA-4 inhibitor) represents a significant advancement in multi-target immune checkpoint blockade strategies. When integrated with RT, this regimen has demonstrated enhanced antitumour efficacy and a manageable safety profile across various malignant STs. In a phase I randomized trial, patients with advanced or unresectable HCC received SBRT followed by either Nivolumab monotherapy or a combination of Nivolumab and Ipilimumab. The dual-agent group demonstrated a favourable trend in ORR, local control and distant metastasis control compared to monotherapy, with an overall manageable toxicity profile (Ref. 178). In HNSCC, a phase I trial investigated the feasibility of combining Nivolumab and Ipilimumab with concurrent RT as an initial curative-intent treatment. The 3-year PFS and OS rates reached 74% and 96%, respectively, demonstrating promising long-term tumour control. However, the study also reported late-onset local toxicities such as soft tissue ulceration in some patients, highlighting the need for improved management of immune-related adverse effects within the irradiated field (Ref. 179). In resectable NSCLC, a clinical study further investigated the addition of Nivolumab and Ipilimumab to CRT. The triplet regimen achieved a pCR rate of 58% and an MPR rate of 73%, markedly surpassing outcomes with conventional CRT. Notably, this enhanced efficacy was not accompanied by a significant increase in perioperative complications, underscoring the regimen’s transformative potential in the neoadjuvant treatment setting (Ref. 182). In patients with NSCLC and brain metastases, a phase 1/2 study evaluated the efficacy and safety of combining Nivolumab and Ipilimumab with SBRT. The results demonstrated that treatment-related grade ≥ 3 AEs occurred at an acceptable rate, with only one case of DLT reported. These findings suggest that this strategy may effectively control intracranial lesions while concurrently supporting systemic immune activation (Ref. 180). Moreover, in a clinical study on NSCLC, Bestvina et al. compared concurrent versus sequential administration of SBRT with Nivolumab plus Ipilimumab. The concurrent treatment arm did not show an increased incidence of severe toxicities and demonstrated promising therapeutic activity. These findings support the potential of combined modality therapy for managing multifocal disease in advanced NSCLC (Ref. 181). However, the efficacy of this combination strategy varies across different tumour types. For instance, in SCLC, clinical studies have shown that consolidation therapy with Nivolumab plus Ipilimumab following standard CRT did not significantly improve PFS or OS. Moreover, a relatively high incidence of irAEs was observed. These findings highlight the need for optimized patient selection and treatment timing, which remain critical areas for future investigation (Ref. 184).

The dual immune checkpoint blockade strategy combining Durvalumab and Tremelimumab is being actively investigated as a potential synergistic partner with RT across a range of STs. Currently, the ‘PD-L1/CTLA-4 dual ICIs combined with RT’ strategy has entered clinical evaluation in immunologically sensitive tumours such as HNSCC and NSCLC, with preliminary data supporting its therapeutic efficacy. In a phase II clinical trial, Kim et al. evaluated the efficacy and safety of combining Durvalumab and Tremelimumab with proton RT in patients with advanced or metastatic HNSCC who had failed multiple prior lines of therapy. The results showed an ORR of 30.4% among 23 evaluable patients, with a median OS of 11.1 months. Notably, abscopal effects were observed in some non-irradiated target lesions, suggesting that this combination strategy may activate a systemic antitumour immune response (Ref. 185). However, the performance of this combination in NSCLC patients has been relatively mixed. A multicentre, open-label phase 2 randomized trial conducted by Schoenfeld et al. enrolled 90 NSCLC patients who had developed resistance to prior PD-L1 inhibitor therapy. The study evaluated the efficacy of three treatment strategies: Durvalumab combined with Tremelimumab alone, Durvalumab plus Tremelimumab with low-dose RT and Durvalumab plus Tremelimumab with HFRT. The results showed comparable ORR across the three groups, with no significant improvement observed in the cohorts receiving RT. These findings suggest that, in the context of PD-L1 inhibitor resistance, the addition of RT does not substantially enhance the therapeutic efficacy of dual ICIs (Ref. 187).

A single-arm phase 2 clinical trial conducted by Stanford University evaluated the efficacy and safety of high-dose precision RT combined with continued ICIs in patients with advanced NSCLC who had progressed after prior PD-1/PD-L1 inhibitor treatment. The study results demonstrated that this combined regimen significantly improved the 24-week PFS rate to 60%, which was statistically superior to the historical control rate of 35% (*p* < 0.001). This study suggests that administering high-intensity local RT in the context of multi-agent ICIs can effectively control progressive lesions and prolong the therapeutic window of systemic ICIs (Ref. 188). Additionally, a randomized phase 2 trial in Belgium further expanded the landscape of multi-target ICIs. The study investigated the combination of SBRT with intravenous Pembrolizumab, intratumoural injections of avelumab and Ipilimumab and CD1c^+^/CD141^+^ myeloid dendritic cells (myDCs) in pretreated patients with oligometastatic STs. Although the study did not meet its primary endpoint, 1-year PFS, some patients achieved clinical benefit, including two partial responses (PR) and two cases of stable disease (SD). These results highlight the feasibility and potential activity of this strategy in selected immunological contexts (Ref. 189).

In addition, Cadonilimab is a novel bispecific ICI that targets both PD-1 and CTLA-4, aiming to achieve dual immune blockade through a single molecule to enhance antitumour immune responses. Compared with conventional dual-agent combination strategies, Cadonilimab is designed to minimize CTLA-4-associated toxicity, offering a broader safety margin. It has demonstrated favourable clinical tolerability and preliminary efficacy across various solid malignancies. In recurrent or metastatic CC, Cadonilimab has been approved by the Chinese National Medical Products Administration (NMPA) as a second-line or later-line therapy. Clinical exploration of its combination with RT is currently underway. Although large-scale prospective trials directly validating the synergy between Cadonilimab and RT are not yet available, several related studies are actively progressing (Supplementary Table 1).

## Challenges and prospects

Although the combination of RT and ICIs has shown promising potential in treating malignant STs, several challenges still need to be addressed. Fundamentally, radioimmunotherapy should be viewed as a dynamic immune modulation process rather than a static combinatorial strategy, in which radiation dose, treatment timing and immune context jointly determine therapeutic outcomes.

From a technical perspective, one major issue is the lack of standardized RT parameters. The optimal dose, fractionation and treatment volume remain uncertain across different tumour types. Preclinical studies suggest that hypofractionated RT may preferentially activate the cGAS-STING pathway and enhance immunogenicity, whereas excessively high single doses induce TREX1 expression, leading to cytosolic DNA degradation and attenuation of immune activation (Ref. 88). These findings highlight the necessity of defining an immunologically optimal radiation window that balances immune stimulation with the risk of immune exhaustion.

Beyond radiation dose itself, the temporal coordination between RT and ICIs represents another critical determinant of therapeutic efficacy. While concurrent delivery may amplify T cell priming and improve tumour control, it is also associated with an increased risk of immune-related toxicity. In contrast, sequential strategies may reduce adverse events but risk missing the optimal window of immunogenic modulation (Refs 56, 58). This suggests that future clinical trial designs should move beyond empirical scheduling and incorporate immune kinetics-guided optimization based on real-time immune profiling.

Even with optimized dose and timing, however, the immunosuppressive TME imposes intrinsic constraints on treatment efficacy. RT can paradoxically recruit Tregs, MDSCs and TAMs, all of which suppress antitumour immunity (Refs 11, 117, 190). Rational integration of microenvironment-targeted interventions, such as TGF-β blockade, chemokine axis inhibition or metabolic reprogramming strategies, may therefore be required to fully unlock the therapeutic potential of radioimmunotherapy (Refs 122, 129).

In this context, accurate patient stratification becomes particularly important. Biomarker development thus represents another critical unmet need. While PD-L1 expression and tumour mutational burden are routinely used in clinical practice, their predictive value remains inconsistent in the context of the combination of RT and ICIs. Multi-dimensional biomarkers integrating immune gene signatures, DNA damage response capacity, microbiota composition and spatial immune architecture may provide more reliable guidance for treatment personalization (Refs 187, 191).

Beyond its ability to enhance antigen release and T-cell priming, RT also dynamically remodels the immune exhaustion landscape through sustained interferon signalling and chronic inflammatory cues. In this context, PD-1/PD-L1 blockade may be insufficient to fully restore antitumour immunity, particularly in tumours exhibiting adaptive or secondary resistance. Targeting alternative inhibitory pathways, such as LAG-3 and TIGIT, in combination with RT, therefore, represents a rational and promising strategy to overcome terminal T cell dysfunction and to extend the durability of immunotherapeutic responses (Refs 48, 53, 89, 92).

As immune intervention becomes increasingly intensive, treatment-related toxicity also emerges as a critical clinical concern. irAEs remain particularly prominent in dual checkpoint blockade or high-dose RT regimens. The development of irAE risk models and adaptive toxicity monitoring strategies will therefore be essential to ensure long-term safety and treatment sustainability (Refs 179, 184).

Looking forward, large-scale randomized trials, molecularly informed treatment algorithms and emerging technologies – such as spatial transcriptomics, AI-assisted radiation planning and longitudinal immune monitoring – are expected to transform the combination of RT and immunotherapy from an empirical approach into a precision-guided immuno-oncology platform.

Ultimately, the integration of RT with ICIs is redefining how local and systemic cancer therapies converge, marking a critical step towards truly mechanism-driven and precision-oriented immuno-oncology.

## Supporting information

10.1017/erm.2026.10041.sm001 Dai et al. supplementary material Dai et al. supplementary material

## Data Availability

The datasets used and/or analysed during the current study are available from the corresponding author on reasonable request.

## Supplementary material

The supplementary material for this article can be found at http://doi.org/10.1017/erm.2026.10041.
