# Supplementary material for: Synergizing Radiotherapy and Immune Checkpoint Inhibitors in Malignant Solid Tumours: Mechanistic Insights and Translational Frontiers
Source: Expert Rev Mol Med. 2026 Mar 10;28:e17. doi: 10.1017/erm.2026.10041 (PMC13148430; doi:10.1017/erm.2026.10041)
Supplement: Dai et al. supplementary material [file S1462399426100416sup001.docx]

**Supplemental table: Ongoing Clinical Trials of RT Combined with ICIs**

| **Disease** | **Trial registration** | **Phase** | **ICIs** | **RT Type** | **Other Treatments** | **Primary Endpoint** |  |
| --- | --- | --- | --- | --- | --- | --- | --- |
| EC | NCT06676449 | Phase 3 | PD-1 inhibitors | TDLN-sparing RT | Paclitaxel, Cisplatin | PFS for patients with TDLN V15 ≤ 50%, 2 years | |
|  |  |  |  |  |  | PFS for all patients, 2 years | |
| HNSCC | NCT06170710 | Phase 2 | PD-1 inhibitors | RT | Cisplatin | DFS, 2 years | |
| NPC | NCT05385926 | Phase 2 | PD-1 inhibitors | RT | Chemotherapy | PFS, 1 year | |
|  |  |  |  | SBRT |  |  |  |
| CSCC | NCT04428671 | Phase 1 | Cemiplimab | RT | Surgery | pCR | |
| MIBC | NCT05203913 | Phase 2 | Nivolumab | RT | Cisplatin, Nab paclitaxel | DFS | |
| HNSCC | NCT03317327 | Phase 1  Phase 2 | Nivolumab | RT | / | Incidence, nature, and severity of AEs graded | |
| SGC | NCT05727410 | Phase 2 | Nivolumab | RT | Docetaxel, Cisplatin; Surgery | Major Pathologic Response rate, 6 months | |
| AC | NCT04046133 | Phase 1 | Pembrolizumab | RT | Mytomycin, Mitomycin, Capecitabine | Safety, 30 days, 6 weeks, 12 weeks, 6 months, 9 months and 12 months | |
|  |  |  |  |  |  | Tolerability, 30 days, 6 weeks, 12 weeks, 6 months, 9 months and 12 months | |
| ACC | NCT06066333 | Phase 2 | Pembrolizumab | Ablation RT | / | AEs, 1 year | |
| BC | NCT04454528 | Phase 1  Phase 2 | Pembrolizumab | HFRT | Surgery | Feasibility, 2 years | |
|  |  |  |  |  |  | Clinical Response, 2 years | |
| BC | NCT03804944 | Phase 2 | Pembrolizumab | Focal RT | CDX-301 | Tolerability, 3 years | |
|  |  |  |  |  |  | Clinical Response rate, 3 years | |
|  |  |  |  |  |  | Pathological Response rate, 3 years | |
| HNSCC | NCT06308913 | Early Phase 1 | Pembrolizumab | Palliative RT | INCB081776 | Incidence of AEs | |
|  |  |  |  |  |  | Duration of AEs | |
|  |  |  |  |  |  | Severity of AEs | |
| HNSCC | NCT04754321 | Phase 1 | Pembrolizumab | EBRT | Surgery | Incidence of AEs, up to 100 days after last dose of study drug | |
|  |  |  |  | Intraoperative RT |  | Health related quality of life, 5 years | |
| HNSCC | NCT04862455 | Phase 2 | Pembrolizumab | HFRT | NBTXR3 | PFS, 2 years | |
|  |  |  |  |  |  | Local failure, 2 years | |
|  |  |  |  | SBRT |  | Regional failure, 2 years | |
|  |  |  |  |  |  | Distant failure, 2 years | |
|  |  |  |  |  |  | ORR, 2 years | |
| HNSCC | NCT04747054 | Phase 3 | Pembrolizumab | Loco-regional RT | Cisplatin, Carboplatin, 5-FU | PFS, disease progression or death (up to 3 years) | |
| GBM | NCT02287428 | Phase 1 | Pembrolizumab | RT | Temozolomide, Personalized NeoAntigen Peptides, Hiltonol | Safety, 2 years | |
|  |  |  |  |  |  | Tolerability, 2 years | |
| NSCLC | NCT05430009 | Phase 1 | Pembrolizumab | SBRT | / | Feasibility, 6 months | |
| RCC | NCT05567588 | Phase 2 | Pembrolizumab | SBRT | / | ORR, 2 months | |
| RCC | NCT05578664 | Phase 2 | Pembrolizumab | RT | Surgery | Relapsed Free Survival, 24 months | |
| Sarcoma | NCT05488366 | Early Phase 1 | Pembrolizumab | SBRT | / | Feasibility, 3 years | |
| TNBC | NCT02977468 | Phase 1 | Pembrolizumab | Intraoperative RT | Surgery | Number of subjects with significant mean percent change in TILs, 3 months | |
| TNBC | NCT04443348 | Phase 2 | Pembrolizumab | Low-dose RT | Paclitaxel, Doxorubicin, Cyclophosphamide, Carboplatin, Capecitabine | TILs | |
|  |  |  |  | High-dose RT |  | Rate of pathologic response in the lymph node | |
| TNBC | NCT04683679 | Phase 2 | Pembrolizumab | SBRT | Olaparib | Overall Response Rate, 8 weeks from baseline | |
| VC | NCT04430699 | Phase 2 | Pembrolizumab | RT | Cisplatin | ORR | |
| SCLC | NCT06350162 | Phase 2 | Serplulimab | RT | / | PFS rate, 1 year | |
| HCC | NCT05366829 | Phase 2 | Tislelizumab | RT | / | PFS rate, 1 year | |
| HNSCC | NCT06725498 | Phase 2 | Tislelizumab | RT | Cisplatin, Paclitaxel | EFS rate, 1 year | |
| HNSCC | NCT05595590 | Phase 2 | Tislelizumab | Pulsed RT | / | ORR, disease progression or death (up to 2 years) | |
|  |  |  |  |  |  | AEs, end of follow-up (up to 2 years) | |
| HCC | NCT06233981 | Phase 2 | Tislelizumab | Moderate-dose HFRT | Lenvatinib | Median (OS), 24 months | |
| NPC | NCT04833257 | Phase 2 | Tislelizumab | RT | Gemcitabine, Cisplatin | CR, 9 weeks | |
| STs | NCT06349837 | Phase 1 | Tislelizumab | Low-dose RT | / | DLT, 24 months | |
|  |  |  |  | SBRT |  |  |  |
| NPC | NCT06455410 | Phase 2 | Adebrelimab | RT | Gemcitabine, Cisplatin | CR rate | |
| HCC | NCT03942328 | Phase 1  Phase 2 | Atezolizumab | EBRT | Bevacizumab, Pneumococcal 13-valent Conjugate Vaccine, Therapeutic Autologous Dendritic Cells | Incidence of significant toxicity, 56 days | |
|  |  |  |  |  |  | PFS rate, 2 years | |
| HCC | NCT06339424 | Phase 2 | Atezolizumab | Photon RT | Bevacizumab | PFS, 12 months | |
| HCC | NCT06133062 | Phase 2 | Atezolizumab | Photon RT | Bevacizumab | PFS, 12 months | |
| HNSCC | NCT01810913 | Phase 2  Phase 3 | Atezolizumab | IMRT | Docetaxel, Cetuximab, Cisplatin | Disease-free survival, 7 years | |
|  |  |  |  |  |  | OS, 7 years | |
| SCLC | NCT06110572 | Phase 1  Phase 2 | Atezolizumab | HFRT | Carboplatin, Etoposide | Incidence of AEs, 30 days | |
|  |  |  |  |  |  | PFS, 6 months | |
| STs | NCT03915678 | Phase 2 | Atezolizumab | RT | BDB001 | CR, PR, and SD, 6 months | |
| BTC | NCT06546969 | Phase 1 | Durvalumab | SFRT | Gemcitabine, Cisplatin, Deep Hyperthermia | Incidence of Grade 3 and higher treatment-related AEs, 90 days post final treatment of Deep Hyperthermia | |
| NSCLC | NCT04748419 | Phase 1  Phase 2 | Durvalumab | HFRT | / | AEs, 2 years | |
|  |  |  |  |  |  | PFS, 12 months | |
| NSCLC | NCT04245514 | Phase 2 | Durvalumab | RT | / | EFS, 12 months | |
| NSCLC | NCT05443971 | Phase 2 | Durvalumab | SFRT | / | Incidence of AEs, 3 months after grid therapy | |
| NSCLC | NCT05128630 | Phase 2 | Durvalumab | HFRT | Cisplatin, Carboplatin, Etoposide | Safety, 0-36 months | |
| RC | NCT04083365 | Phase 2 | Durvalumab | RT | Capecitabine, Surgery | pCR rate | |
| SCLC | NCT04728230 | Phase 1  Phase 2 | Durvalumab | RT | Carboplatin, Etoposide, Olaparib | Incidence of DLT | |
| SCLC | NCT05796089 | Phase 2 | Durvalumab | Thoracic RT | Cisplatin, Carboplatin, Etoposide | Safety, 90 days | |
|  |  |  |  |  |  | Feasibility, 90 days | |
| SCLC | NCT06406673 | Phase 2 | Cadonilimab | Palliative RT | Cisplatin, Carboplatin, Etoposide, Vorolanib | ORR, 24 months | |
| HNSCC | NCT06494995 | Phase 2 | Cadonilimab | Low-dose RT | Capecitabine | ORR, 2 years | |
|  |  |  |  | SBRT |  |  |  |
| NPC | NCT05941741 | Phase 3 | Cadonilimab | IMRT | Gemcitabine; Cisplatin | PFS rate, 3 year | |
| ADC | NCT03589339 | Phase 1 | Pembrolizumab | SBRT | NBTXR3 | Determination of the Recommended Dose, 24 months | |
|  |  |  | Nivolumab |  |  | Incidence of Grade 3 and higher treatment-related AEs, 24 months | |
| HCC | NCT04430452 | Phase 2 | Durvalumab | HFRT | / | ORR, 24 months | |
|  |  |  | Tremelimumab |  |  |  |  |
| NSCLC | NCT03391869 | Phase 3 | Ipilimumab | RT | Surgery | OS, 1 year; | |
|  |  |  | Nivolumab |  |  |  |  |
| NPC | NCT06675214 | Phase 3 | Adebrelimab | Standard-dose IMRT,  Gradient Fractionated IMRT | Cisplatin | PFS, 3 year | |
|  |  |  | Camrelizumab |  |  |  |  |
|  |  |  | Toripalimab |  |  |  |  |
| NPC | NCT06092957 | Phase 3 | Adebrelimab | Standard-dose IMRT, Reduced-dose IMRT | Cisplatin | PFS, 3 year | |
|  |  |  | Camrelizumab; |  |  |  |  |
|  |  |  | Toripalimab |  |  |  |  |

**Abbreviations:**

1. Anal Cancer (AC), Adrenocortical Cancer (ACC), Advanced Cancers (ADC), Breast Cancer (BC), Biliary Tract Cancer (BTC), Cutaneous Squamous Cell CCarcinoma (CSCC), Esophageal Cancer (EC), Glioblastoma (GBM), Hepatocellular Cancer (HCC), Head and Neck Squamous Cell Cancer (HNSCC), Muscle Invasive Bladder Cancer (MIBC), Nasopharyngeal Cancer (NPC), Non-Small Cell Lung Cancer (NSCLC), Rectal Cancer (RC), Renal Cell Cancer (RCC), Salivary Gland Cancer (SGC), Small Cell Lung Cancer (SCLC), Solid Tumors (STs), Triple Negative Breast Cancer (TNBC), Vulvar Cancer (VC)
2. External Beam Radiation Therapy (EBRT), Hypofractionated Radiation Therapy (HFRT), Intensity Modulated Radiation Therapy (IMRT), Radiation Therapy (RT), Stereotactic Body Radiation Therapy (SBRT), Spatially Fractionated Radiation Therapy (SFRT), Tumor Draining Lymph Nodes-sparing Radiation Therapy (TDLN-sparing RT)
3. 5-fluorouracil (5-FU)
4. Adverse Events (AEs), Complete Response, (CR), Disease Free Survival (DFS), Dose Limiting Toxicity (DLT), Event-free survival (EFS), Objective Response Rate (ORR), Overall Survival (OS), Progression-free Survival (PFS), Partial Response (PR), Pathological Complete Response (pCR), Stable Disease (SD), Tumor-Infiltrating Lymphocytes (TILs)
